# P21. NK-cell dysfunction in human renal carcinoma reveals diacylglycerol kinase as key regulator and target for therapeutic intervention

**DOI:** 10.1186/2051-1426-2-S2-P12

**Published:** 2014-03-12

**Authors:** P Prinz, E Noessner

**Affiliations:** 1 Helmholtz Zentrum Muenchen, Molecular Immunology, Munich, Germany

## 

NK cells are appreciated as antitumour effector cells in mouse models and human hematologic malignancies but their relevance in immunosurveillance of human solid tumours remains conflicting due to problems with *in situ* detection and reports of functional inactivity in the tumour milieu. The study was performed to identify mechanisms that impair NK-cell function in the tumour milieu and thereby identify therapeutic targets that allow recovery of NK-cell functionality.

We used *in situ* detection and flow cytometry to localise, quantify and profile NK cells of human clear cell renal cell carcinoma (ccRCC) tissue. Strategies were evaluated to reinstate functionality of tumour-derived NK cells. *In vitro* coculture models were applied to gain mechanistic insight into tumour-induced NK-cell alterations.

Tumour-resident NK cells, compared to NK cells from non-tumour kidney and PBLs, displayed conjoint phenotypic alterations and dysfunction induced by the tumour milieu, which were associated mechanistically with high levels of signaling attenuator diacylglycerol kinase (DGK)-a and blunted mitogen-activated protein kinase pathway activation (ERK1/2, JNK). Reinstating NK-cell functionality was possible by DGK-inhibition or brief IL-2-culture, interventions that de-repressed the ERK pathway. The extent of alteration and magnitude of recovery could be linked to NK-cell frequency within ccRCC-infiltrating lymphocytes, possibly explaining the observed survival benefit of patients with NK^high^ tumours.

DGK-mediated dampening of the ERK pathway ensuing in NK-cell dysfunction was identified as an important escape mechanism in ccRCC. DGK and the ERK pathway emerge as promising therapeutic targets to restore suppressed NK-cell activity for the improvement of antitumour immunity.

